# Accuracy of reporting of Aboriginality on administrative health data collections using linked data in NSW, Australia

**DOI:** 10.1186/s12874-020-01152-2

**Published:** 2020-10-28

**Authors:** Michael A. Nelson, Kim Lim, Jason Boyd, Damien Cordery, Allan Went, David Meharg, Lisa Jackson-Pulver, Scott Winch, Lee K. Taylor

**Affiliations:** 1 Centre for Epidemiology and Evidence, NSW Ministry of Health, Sydney, NSW Australia; 2 Bureau of Health Information, Chatswood, NSW Australia; 3 System Information and Analytics, NSW Ministry of Health, Sydney, NSW Australia; 4 Faculty of Medicine and Health, Sydney School of Health Sciences and the Poche Centre for Indigenous Health, University of Sydney, Sydney, NSW Australia; 5 Deputy Vice-Chancellor, Indigenous Strategy and Services, University of Sydney, Sydney, Australia; 6 Scott Winch, World Vision, Sydney, Australia

**Keywords:** Aboriginal health, Indigenous health, Administrative data, Linked data, Data linkage, Record linkage, Reporting, Identification

## Abstract

**Background:**

Aboriginal people are under-reported on administrative health data in Australia. Various approaches have been used or proposed to improve reporting of Aboriginal people using linked records. This cross-sectional study used self-reported Aboriginality from the NSW Patient Survey Program (PSP) as a reference standard to assess the accuracy of reporting of Aboriginal people on NSW Admitted Patient (APDC) and Emergency Department Data Collections (EDDC), and compare the accuracy of selected approaches to enhance reporting Aboriginality using linked data.

**Methods:**

Ten PSP surveys were linked to five administrative health data collections, including APDC, EDDC, perinatal, and birth and death registration records. Accuracy of reporting of Aboriginality was assessed using sensitivity, specificity, and positive and negative predictive values (PPVs and NPVs) and F score for the EDDC and APDC as baseline and four enhancement approaches using linked records: “Most recent linked record”, “Ever reported as Aboriginal”, and two approaches using a weight of evidence, “Enhanced Reporting of Aboriginality (ERA) algorithm” and “Multi-stage median (MSM)”.

**Results:**

There was substantial under-reporting of Aboriginality on APDC and EDDC records (sensitivities 84 and 77% respectively) with PPVs of 95% on both data collections. Overall, specificities and NPVs were above 98%. Of people who were reported as Aboriginal on the PSP, 16% were not reported as Aboriginal on any of their linked records. Record linkage approaches generally increased sensitivity, accompanied by decrease in PPV with little change in overall F score for the APDC and an increase in F score for the EDDC. The “ERA algorithm” and “MSM” approaches provided the best overall accuracy.

**Conclusions:**

Weight of evidence approaches are preferred when record linkage is used to improve reporting of Aboriginality on administrative health data collections. However, as a substantial number of Aboriginal people are not reported as Aboriginal on any of their linked records, improvements in reporting are incomplete and should be taken into account when interpreting results of any analyses. Enhancement of reporting of Aboriginality using record linkage should not replace efforts to improve recording of Aboriginal people at the point of data collection and addressing barriers to self-identification for Aboriginal people.

**Supplementary Information:**

The online version contains supplementary material available at 10.1186/s12874-020-01152-2.

## Background

Accurate recording of Aboriginal people on population health administrative data collections is essential to correctly measure the health gap between Aboriginal and non-Aboriginal people and to monitor and evaluate programs that aim to reduce health disparities. The Australian National Best Practice Guidelines for Collecting Indigenous Status in Health Data Sets requires patients to be asked a standard question at every health system encounter, allowing individuals to respond differently at each contact [1]. Although the quality of health information on Aboriginal people has improved in Australia over time, administrative data collections continue to underestimate the true number of Aboriginal people that utilise health services [2]. In 2011–12, 80% of Aboriginal patients were estimated to be correctly reported on NSW hospital records [3].

While efforts continue to improve reporting of Aboriginal people on administrative data collections in Australia [4], various approaches have been proposed or used to enhance reporting of Aboriginal people on administrative data collections using record linkage, including:

- ever reported: a person is recorded as Aboriginal on any linked record [5–13]
- always reported: a person is recorded as Aboriginal on all linked records [7, 8]
- index record: a person is recorded as Aboriginal on first record in the chronological series [8, 9]
- most recent record: a person is recorded as Aboriginal on the last record in the chronological series [11–13]
- at least two hospitals: a person is recorded as Aboriginal at more than one hospital [11]
- majority of records: recorded as Aboriginal on at least 50% of all public hospital admissions [8, 9, 12]
- Enhanced reporting of Aboriginality (ERA) algorithm: if the person has 3 or more independent sources of information on the linked dataset, at least 2 must indicate that the person is Aboriginal; if the person has 1 or 2 independent sources of information, 1 is sufficient to consider the person to be Aboriginal [5, 12–15]
- Multi-stage median: each person is given a derived Aboriginal status for each data collection in the linked dataset, and the collection-derived Aboriginal status is combined into an overall derived Aboriginal status for each person [7, 11, 13,14]

When comparing the performance of the different approaches, most studies report the percentage change in number of records reported as Aboriginal following enhancement compared to the original reported value. This comparison does not take into account the accuracy of the enhancement. To date, no published studies have assessed the accuracy of approaches to improve reporting of Aboriginal people using an independent reference standard.

In this study, we used a New South Wales (NSW) Patient Survey Program (PSP) dataset as an external reference standard. We used the PSP as an external reference standard for reporting of Aboriginal people due to the voluntary nature of participation, the completion of the survey in a person’s own time and in the privacy of his/her own home, and advice to participants that individual responses are not accessible to health care providers.

This study uses self-reported Aboriginality from the NSW Patient Survey Program to:

1. assess the accuracy of reporting of Aboriginal people on NSW hospital and emergency department data collections, and
2. compare the accuracy of a range of approaches to enhance reporting of Aboriginal people on NSW hospital and emergency department data collections.

## Methods

### Study design

Cross-sectional observational study using linked population health administrative data.

### Study population

Patients who were admitted to hospital or attended an emergency department in 2013–2015, completed a relevant PSP survey and gave consent for record linkage.

### Aboriginal people

We use the term “Aboriginal people” to refer to both Aboriginal and Torres Strait Islander peoples for the purpose of this study.

### Data sources

The PSP collects information on the experiences of people who have recently had contact with the NSW public health system to facilitate performance reporting on patient satisfaction with health services. The PSP is managed by the NSW Bureau of Health Information (BHI) [15]. Since 2013, the PSP has sought consent for the information to be used for research, including record linkage studies.

Ten PSP datasets were included in this study: Adult Admitted Patient Survey (2013, 2014 and 2015), Emergency Department Patient Survey (2013–14, 2014–15 and 2015–16), Admitted Children and the Young Patients Survey (2015), Small and Rural Hospitals Survey (2015), Small Hospital Emergency Care Survey (2015–16) and the Maternity Care Survey (2015). Of the 181,747 respondent records in the ten PSP datasets, 150,452 (83%) included consent for record linkage; there was no difference in the consent rates between Aboriginal and non-Aboriginal people. The number of records in each PSP dataset varied from 4128 to 35,962 and the proportion of respondents that consented varied from 79 to 90% (Table S1).

The ten PSP datasets were linked to the following administrative datasets: the NSW Perinatal Data Collection (PDC), NSW Admitted Patient Data Collection (APDC), NSW Emergency Department Data Collection (EDDC), Registry of Births, Deaths and Marriages birth registrations (RBDM Births) and the Cause of Death Unit Record File (CODURF).

### Record linkage and dataset preparation

The 150,452 PSP records for consenting participants were linked by the NSW Centre for Health Record Linkage (CHeReL) [16] to records of the APDC, EDDC, RBDM Births (as mother, baby or other parent), PDC (as mother or baby) and CODURF, within 2 years of the PSP survey dates (2011 to 2016–17). Of the 150,452 linked PSP records, 2996 (2.2%) were excluded due to missing information (*n* = 2890) or conflicting information on Aboriginality across PSP datasets (*n* = 106).

The final linked dataset for analysis comprised 1,265,799 linked records relating to 130,514 persons: 75041 records for Aboriginal people (5102 Aboriginal people) and 1,190,758 records for non-Aboriginal people (125,412 non-Aboriginal people). The contribution of source dataset records to the linked dataset was: APDC, *n* = 714,704 (56.5%); EDDC, *n* = 515,907 (40.8%); RBDM birth registration records (babies and parents), *n* = 16,387 (1.3%); PDC records (babies and mothers), *n* = 14,377 (1.1%); and CODURF, *n* = 4424 (0.3%).

### Statistical analysis

We estimated the level of reporting of Aboriginal people on the APDC and EDDC by comparing Aboriginality reported to the PSP with Aboriginality as recorded on the APDC or EDDC record that was originally sampled for the PSP. In addition to this “As-recorded” measure, we compared four approaches to enhance reporting of Aboriginality using linked records of the PDC, APDC, EDDC, RBDM Births and COD URF datasets for all events for the person:

1. “Most recent”: Aboriginality reported at the most recent admission/presentation
2. “ERA algorithm”: The ERA algorithm is a weight of evidence approach that relies on independent sources of information. Each independent report is counted as a “unit of information” that contributes to the weight of evidence as to whether a person is reported as Aboriginal:

i) if the person has 3 or more units of information, at least 2 indicating that the person is Aboriginal or Torres Strait Islander; or
ii) if the person has 1 or 2 units of information, 1 is sufficient to report the person as Aboriginal or Torres Strait Islander.

3. “Multi-stage median” (MSM): The MSM is a weight of evidence approach that applies the ERA algorithm within each data collection and then applies the ERA algorithm a second time using the results from each data collection as the unit of information.
4. “Ever reported”: A single linked record from any dataset is sufficient to report a person as Aboriginal or Torres Strait Islander.

We calculated measures of validity, including sensitivity, specificity, positive predictive value (PPV), negative predictive value (NPV), and F score for “As-recorded” Aboriginality and for each of the 4 enhancement methods using the PSP as the standard. Measures of validity were calculated for the APDC and EDDC separately. For each data source the accuracy of the “As-recorded” and enhancement algorithms are described overall and by age.

## Results

Of the 130,514 people in the PSP study population, 5102 (3.9%) reported themselves to be Aboriginal. Compared to non-Aboriginal people, Aboriginal people in the study population had a similar sex distribution, were substantially younger and more likely to live in a non-metropolitan area (Table 1).

**Table 1:** Demographic characteristics of PSP study population

Characteristic	Total		Aboriginal		Non-Aboriginal	
	No.	%	No.	%	No.	%
Total	130,514	100.0	5102	100.0	125,412	100.0
Sex						
Male	60,332	46.2	2334	45.7	57,998	46.2
Female	70,178	53.4	2768	54.3	67,410	53.4
Age (years)						
0–19	18,511	14.2	866	17.0	17,645	14.1
20–39	18,527	14.2	746	14.6	17,781	14.2
40–64	41,222	31.6	2216	43.4	39,006	31.1
65+	52,254	40.0	1274	25.0	50,980	40.7
Remoteness Area						
Major cities	71,337	54.6	2159	42.3	69,178	55.2
Inner regional	45,400	34.8	2162	42.4	43,238	34.5
Outer regional	13,022	10.0	684	13.4	12,338	9.8
Remote and very remote	755	0.6	97	1.9	658	0.5

Of the 5102 Aboriginal people reported on the PSP: 272 (5%) had 1 linked record; 448 (9%) had 2 linked records; and 4382 (86%) had 3 or more linked records. Of the 5102, 838 (16%) had no linked record that recorded the person as Aboriginal; these 838 people related to 3314 linked APDC records and 3440 linked EDDC records for Aboriginal people (8 and 11% respectively).

Of the 125,412 non-Aboriginal people reported on the PSP: 12458 (10%) had 1 linked record; 15,427 (12%) had 2 linked records; and 97,527 (78%) had 3 or more linked records. Of the 125,412 non-Aboriginal people reported on the PSP: 677 (0.5%) were recorded as Aboriginal on one or more of their linked records; 120 (0.1%) were consistently reported as Aboriginal (44 had 1 linked record; and 633 had 2 or more linked records, of which 76 were consistently reported as Aboriginal); 508 (0.05%) were considered Aboriginal by the ERA approach; and 497 (0.05%) were considered Aboriginal by MSM approach.

Using the PSP as the reference, the overall sensitivity, PPV and F score of Aboriginal people “As-recorded” on the APDC were 84, 95% and 0.90 respectively, and on the EDDC were 77, 95% and 0.85 respectively (Table 2). Specificities and NPVs for both APDC and EDDC and were above 98%. When age groups were compared, the sensitivity, PPV and F scores of Aboriginal people “As-recorded” were highest among 40–64 year olds for both the APDC and EDDC, and lowest among 20–39 year olds in the APDC and 0–19 year olds in the EDDC. Specificities and NPVs were generally over 98%.

**Table 2:** Record-level measures of validity by data source, age and reporting method

Age	Method	APDC					EDDC				
**(years)**		**Sensitivity**	**Specificity**	**PPV**	**NPV**	$\mathcal{F}$	**Sensitivity**	**Specificity**	**PPV**	**NPV**	$\mathcal{F}$
		**%**	**%**	**%**	**%**		**%**	**%**	**%**	**%**	
0–19	As recorded	75.1	99.7	93.6	98.6	0.83	72.2	99.6	93.5	98.0	0.81
	Enhanced:										
	1. Most recent	79.9	99.7	94.4	98.9	0.87	80.1	99.6	93.4	98.5	0.86
	2. ERA algorithm	83.0	99.4	88.9	99.0	0.86	82.5	99.2	88.5	98.7	0.85
	3. Multi-stage median	82.3	99.5	89.6	99.0	0.86	81.7	99.3	89.1	98.6	0.85
	4. Ever reported	87.8	99.1	85.2	99.3	0.87	85.3	98.9	85.0	98.9	0.85
20–39	As recorded	72.5	99.6	91.7	98.2	0.81	75.8	99.4	92.3	97.8	0.83
	Enhanced:										
	1. Most recent	68.5	99.5	91.0	97.9	0.78	81.3	99.2	90.3	98.3	0.86
	2. ERA algorithm	83.4	99.3	89.0	98.9	0.86	89.7	98.8	87.4	99.0	0.89
	3. Multi-stage median	82.9	99.4	89.9	98.9	0.86	89.2	98.9	88.2	99.0	0.89
	4. Ever reported	85.0	99.1	86.4	99.0	0.86	91.5	98.6	85.6	99.2	0.88
40–64	As recorded	88.0	99.8	97.5	98.8	0.93	81.0	99.7	96.5	97.9	0.88
	Enhanced:										
	1. Most recent	88.4	99.8	97.8	98.8	0.93	82.3	99.6	96.3	98.1	0.89
	2. ERA algorithm	93.9	99.2	92.2	99.4	0.93	91.2	99.2	92.7	99.0	0.92
	3. Multi-stage median	93.9	99.4	94.6	99.4	0.94	91.3	99.2	92.9	99.0	0.92
	4. Ever reported	94.6	99.0	91.1	99.4	0.93	92.4	99.0	91.4	99.2	0.92
65+	As recorded	83.6	99.8	93.8	99.3	0.88	75.1	99.8	93.4	99.1	0.83
	Enhanced:										
	1. Most recent	80.9	99.7	91.3	99.2	0.86	77.0	99.8	94.6	99.2	0.85
	2. ERA algorithm	89.9	99.3	84.2	99.6	0.87	84.7	99.5	87.1	99.5	0.86
	3. Multi-stage median	89.7	99.3	84.5	99.6	0.87	84.4	99.6	88.1	99.4	0.86
	4. Ever reported	90.9	99.1	81.9	99.6	0.86	85.4	99.4	83.5	99.5	0.84
Total	As recorded	84.4	99.7	95.3	99.0	0.90	77.4	99.7	94.7	98.5	0.85
	Enhanced:										
	1. Most recent	83.2	99.7	94.4	99.0	0.88	80.4	99.7	94.5	98.7	0.87
	2. ERA algorithm	90.9	99.3	88.3	99.4	0.90	88.0	99.3	89.8	99.2	0.89
	3. Multi-stage median	90.7	99.3	89.5	99.4	0.90	87.7	99.4	90.4	99.2	0.89
	4. Ever reported	92.0	99.1	86.4	99.5	0.89	89.3	99.1	87.4	99.3	0.88

Using the PSP as the reference and comparing to the “As-recorded” approach, the “ERA Algorithm”, “MSM” and “Ever reported” enhancement methods produced overall increases in sensitivity though at the cost of a decrease in PPV. The “most recent” method produced a lower sensitivity and PPV for the APDC, and a slightly higher sensitivity and equivalent PPV for the EDDC. Specificities and NPVs were generally over 98%. In terms of a balance between maximising sensitivity and minimising the accompanying reduction in PPV: for the APDC, F scores were similar across all enhancement methods, no enhanced method resulted in a higher F score than “As-recorded”, though the “MSM” and “ERA algorithm” methods produced an equivalent F score of 0.90; and for the EDDC, all enhanced methods produced a higher F score, with “MSM” and “ERA algorithm” methods producing the highest F score of 0.89. When age groups were examined, similar patterns were observed with the possible exception of the youngest age group (0–19 years), where the “Most recent” enhancement approach produced a very marginally better F score associated with a relatively higher PPV.

## Discussion

This is the first published study to quantify the level of reporting of Aboriginal people on administrative health data collections using an external reference standard and validates a range of methods using linked data to enhance reporting. Using the PSP as a reference standard, we found that the sensitivity and PPV of reporting of Aboriginal people on the APDC was 84 and 95% respectively, and on the EDDC was 77 and 95% respectively. Specificities and NPVs were generally over 98% while F scores were generally above 0.85. Importantly, we found that 16% of people who were reported as Aboriginal on the PSP were not reported as Aboriginal on any of their linked records. This is similar to the results of a recent Queensland study that examined ED presentations in a single facility [17].

Of the four enhancement methods examined in this study, the “ERA algorithm” and “MSM” approaches had the overall highest F scores (APDC: 0.90, EDDC: 0.89) and improved the sensitivity of reporting of Aboriginal people compared to an “As-recorded” approach at the cost of decreased PPV for both the APDC and EDDC, with overall sensitivities of 91% and PPVs of 88–89% for the APDC, and sensitivities of 88% and PPVs of 90% for the EDDC.

The “ERA algorithm” and “MSM” approaches take account of the weight of evidence that a person is Aboriginal and offset the possibility of incorrect enhancement due to administrative health records being incorrectly reported as relating to an Aboriginal person, or to incorrectly linked records. The CHeReL has a range of approaches to record linkage [18]. The probabilistic linkage procedure used by the CHeReL for this project was designed to achieve a false positive rate of no more than 0.5% [19].

The “MSM” approach takes into account the possibility of systematic differences in patterns of reporting of Aboriginality in different data collections, and that data collections may vary greatly in the number of records that they hold. It is argued that if these issues are not taken into account, then an enhancement approach may ultimately only reflect Aboriginality as reported in whichever collection has the most records about a person [3]. In this study, the “MSM” and “ERA algorithm” approaches had the same overall F scores, and similar PPVs and sensitivities for both the APDC and EDDC.

The “Ever-reported” approach had the highest sensitivity and lowest PPV of all enhancement methods. The relatively low PPV demonstrates the vulnerability of the method to incorrect reporting of non-Aboriginal people as Aboriginal. The “Most-recent” method had the lowest sensitivity of all the approaches, and in the case of the APDC, lower sensitivity than the “As-recorded” approach.

There were 677 PSP respondents who were reported as non-Aboriginal on the PSP and reported as Aboriginal on at least one linked administrative health record, with 508 of these meeting one of the weight of evidence criteria for reporting a person as Aboriginal and 120 consistently reported as Aboriginal on all their linked records. Under-reporting and inconsistencies in reporting of Aboriginal people on administrative health data collections may be due to health staff not asking patients about their Aboriginality or Aboriginal people choosing not to self-report in a particular context. Non-Aboriginal people may be incorrectly reported as Aboriginal on administrative health data collections by health staff mistakenly reporting indigenous peoples from other countries as Aboriginal.

The strengths of our study are that it is population-based, and that the PSP datasets are representative samples from relevant public hospital and emergency department datasets for each patient survey and are independent sources of information on self-reported Aboriginality. The limitations of the study are:

- The PSP is not perfect reference standard. The PSP was endorsed as the reference standard by the project Aboriginal Reference Group due to the exclusive self-report approach and the safe context, that is, the voluntary nature of participation, completion of the survey in a person’s own time and in the privacy if their own home, and advice to participants that individual responses are not accessible to health care providers. Our finding that 16% (*n* = 838) of people who were reported as Aboriginal on the PSP were not reported as Aboriginal on any of their linked records tends to favour the use of the PSP as a reference standard. The small proportion of PSP records (2.2%) excluded due to missing information on Aboriginality or conflicting information across PSP datasets indicates that the PSP is not a perfect reference standard. The approximately 500 (0.05%) people who were reported as non-Aboriginal on the PSP and met one of the weight of evidence criteria on their linked administrative records also suggests that the PSP is not a perfect reference standard; however this finding could also represent incorrect or inconsistent reporting of Aboriginal people across the APDC and EDDC datasets, or false positive links. A less than perfect sensitivity of the PFP would impact on the results; in particular, the PPVs and F scores of the APDC and EDDC would be underestimated in this study.
- Incorrect links may also contribute to inconsistent reporting across linked records. Incorrect links are more likely within families or households where names or addresses are similar. Where families and households comprise a mix of Aboriginal and non-Aboriginal people, incorrect links may result in apparent inconsistency in reporting of a person’s Aboriginality.
- Data linkage was limited to a window of two years before and after the PSP in accordance with patient consent—a longer time period would have increased the number of linked records and increased the potential for further enhancement of reporting of Aboriginality.
- The combined PSP surveys included in the study population represent a sampling frame for the study population that may not be representative of APDC and EDDC records generally. The sampling frame for nine of the 10 surveys were adults, with only one survey (2% of total PSP records) targeted at children and young people. Also, this study was based on a linked dataset derived from a cohort of people attending public hospitals and emergency departments; private hospital admissions account for 39% of hospital activity in NSW.
- Previous comparisons of weight of evidence approaches [4, 6] have shown that using information from linked records to enhance reporting of Aboriginality reduces the number of records with missing data, improves consistency within records for individuals and increases the overall number of records classified as Aboriginal. By using the PSP as a reference, we found that where an enhancement approach increases sensitivity, that is, increases the proportion of records correctly classified as relating to Aboriginal people, PPV is decreased by increasing the proportion of records incorrectly classified as relating to Aboriginal people, with no change in the overall F score for the APDC and an increase in F score for the EDDC.

We found that 16% of people who were reported as Aboriginal on the PSP were not reported as Aboriginal on any of their linked records. These 16% of people represent 8% of linked APDC and 11% of linked EDDC records for Aboriginal people in the study. This creates an absolute limit on the potential for record linkage to enhance reporting of Aboriginal people on these datasets. Of the approaches tested, we found that the weight of evidence approaches, “ERA algorithm” and “MSM”, performed best. Inclusion of more years of data in the linkage is likely to improve the enhancement. Consideration of family linkages may improve the reporting of Aboriginal children [14]. Inclusion of a greater range of administrative datasets in the linkage may also improve the enhancement; however it is important to bear in mind that contributing data sources must collect information on Aboriginality independently of each other in order to contribute to the weight of evidence.

## Conclusion

Enhanced reporting of Aboriginal people using record linkage does not define a person as Aboriginal. It is a statistical construct that results in improved information about Aboriginal people for the purposes of planning and managing health services. Weight of evidence approaches are preferred when record linkage is used to improve reporting of Aboriginality on administrative health data collections. However, even the most accurate enhancement approaches substantially under-report Aboriginal people on administrative datasets and this should be taken into account in the interpretation of results of any analyses. These results highlight the importance of continued efforts to improve recording of Aboriginal people on administrative data at the point of data collection and addressing barriers to self-identification for Aboriginal people.

## Supplementary Information

**Additional file 1.** Table S1 PSP respondents and consent for data linkage by Aboriginality

## Data Availability

The datasets generated during the study are not publicly available as they contain information that could potentially re-identify individuals; however, datasets are available from the corresponding author on reasonable request and with relevant ethical approval.

## Abbreviations

APDC: Admitted Patient Data Collection
BHI: Bureau of Health Information
CHeReL: Centre for Health Record Linkage
CODURF: Cause of Death Unit Record File
EDDC: Emergency Department Data Collection
ERA: Enhanced Reporting of Aboriginality
MSM: Multi-stage Median
NPV: Negative Predictive Value
NSW: New South Wales
PDC: Perinatal Data Collection
PPV: Positive Predictive Value
PSP: Patient Survey Program
RBDM: Registry of Births, Deaths and Marriages
