# Supplementary material for: Accuracy of reporting of Aboriginality on administrative health data collections using linked data in NSW, Australia
Source: BMC Med Res Methodol. 2020 Oct 28;20:267. doi: 10.1186/s12874-020-01152-2 (PMC7594479; doi:10.1186/s12874-020-01152-2)
Supplement: Supplementary file 1 — Additional file 1. Table S1 PSP respondents and consent for data linkage by Aboriginality [file 12874_2020_1152_MOESM1_ESM.docx]

**Supplementary Tables**

**Table S1 PSP respondents and consent for data linkage by Aboriginality**

| **Survey** | **Aboriginal** | | | **Non-Aboriginal** | | | **Overall** | | |
| --- | --- | --- | --- | --- | --- | --- | --- | --- | --- |
|  | **Respondents** | **Consent for data linkage** | | **Respondents** | **Consent for data linkage** | | **Respondents** | **Consent for data linkage** | |
|  | **No.** | **No.** | **%** | **No.** | **No.** | **%** | **No.** | **No.** | **%** |
| Adult Admitted Patient 2013 | 802 | 659 | 82 | 35 160 | 29 951 | 85 | 35 962 | 30 610 | 85 |
| Adult Admitted Patient 2014 | 2 682 | 2 321 | 87 | 24 029 | 20 659 | 86 | 26 711 | 22 980 | 86 |
| Adult Admitted Patient 2015 | 651 | 575 | 88 | 27 740 | 23 925 | 86 | 28 391 | 24 500 | 86 |
| Small and Rural Hospitals 2015 | 216 | 164 | 76 | 6 592 | 5 266 | 80 | 6 808 | 5 430 | 80 |
| Emergency Department 2013-14 | 668 | 513 | 77 | 25 186 | 20 001 | 79 | 25 854 | 20 514 | 79 |
| Emergency Department 2014-15 | 472 | 365 | 77 | 17 829 | 13 854 | 78 | 18 301 | 14 219 | 78 |
| Emergency Department 2015-16 | 652 | 488 | 75 | 23 158 | 18 223 | 79 | 23 810 | 18 711 | 79 |
| Small Hospital Emergency Care 2015 | 356 | 294 | 83 | 6 687 | 5 509 | 82 | 7 043 | 5 803 | 82 |
| Maternity Care Survey 2015 | 134 | 119 | 89 | 4 605 | 4 155 | 90 | 4 739 | 4 274 | 90 |
| Child and Young Person 2014 | 425 | 363 | 85 | 7 968 | 6 843 | 86 | 8 393 | 7 206 | 86 |
| Total | 7 058 | 5 861 | 83 | 178 954 | 148 386 | 83 | 186 012 | 154 247 | 83 |
